# Evaluation of bioavailable ^137^Cs transfer from forest litter to Scarabaeidae beetle (*Protaetia orientalis*) through a breeding experiment in Fukuhshima

**DOI:** 10.1371/journal.pone.0310088

**Published:** 2024-09-06

**Authors:** Jaeick Jo, Yumiko Ishii, Rie Saito, Asuka Tanaka, Seiji Hayashi

**Affiliations:** 1 Environmental Impact Assessment Section, Fukushima Branch, National Institute for Environmental Studies, Miharu, Fukushima, Japan; 2 Savannah River Ecology Laboratory, University of Georgia, Aiken, South Carolina, United States of America; 3 Department of Animal Science, Faculty of Agriculture, Iwate University, Morioka, Iwate, Japan; Universiti Teknologi Malaysia, MALAYSIA

## Abstract

Following the Fukushima Daiichi Nuclear Power Plant accident in 2011, most of the released ^137^Cs remained in the litter and surface soil of the adjacent forest floor. However, ^137^Cs absorption by large soil invertebrates near this site has not been estimated. The aim of this study was to understand the role of soil macroinvertebrates in ^137^Cs uptake from forest litter into forest ecosystems. Breeding experiments were conducted using scarab beetle larvae (*Protaetia orientalis*). Dissection experiments revealed that 85% of the total ^137^Cs was concentrated in the digestive tract of larvae, while a low proportion was absorbed into the skin and muscle tissues. The ^137^Cs absorption rate, indicating the transfer of ^137^Cs from consumed litter to larval tissue, was low (0.39%). ^137^Cs concentrations decreased to one-fourth from larva to imago, possibly due to excretion from the digestive tract and during eclosion. In the elimination experiment, biological half-lives were 0.26–0.64 and 0.11–0.47 days and 3.35–48.30 and 4.01–17.70 days for the digestive tract and muscle/skin tissues in the fast and slow components, respectively, corresponding to ^137^Cs discharge from the gastrointestinal tract and physiological clearance. In the sequential extraction experiment, litter digestion by flower chafer larvae significantly reduced the bioavailable fraction of ^137^Cs including water-soluble, exchangeable, oxidized, and organic forms, from 23.2% in litter to 17.7% in feces. Residual ^137^Cs was not reduced by digestion, probably because it was fixed in soil clay. Our study on breeding experiments of the Scarabaeidae beetle confirmed the low bioavailability of ^137^Cs in the litter in Fukushima. However, litter feeders may play an important role in transferring ^137^Cs to higher trophic levels in the forest ecosystem by extracting the bioavailable fraction of the vast stock of ^137^Cs on the forest floor.

## Introduction

The Fukushima Daiichi Nuclear Power Plant accident that occurred in March 2011 released large quantities of radiocesium (^137^Cs and ^134^Cs) into the environment. Because the half-life of ^137^Cs (30.2 yr) is longer than that of ^134^Cs (2.06 yr), it persists for a longer time [1]. In forested areas, ^137^Cs is initially trapped in the leaves and branches of the forest canopy and subsequently migrates downward to the forest floor through litterfall and rain [2, 3]. Most of the total ^137^Cs stays in the upper part of the mineral soil layer (0–5 cm depth) [4–6] because ^137^Cs is fixed on frayed edge sites in the interlayers of illite and micaceous clay minerals. This retention is nearly irreversible and limits the vertical migration of ^137^Cs into deeper forest soil layers [7]. However, some ^137^Cs remains in a bioavailable form in the surface forest floor layer for a long time because of the cycling process of uptake and defoliation by trees and ^137^Cs accumulation by fungal activities [8–11]. ^137^Cs in organic matter can serve as a major source of ^137^Cs cycling in the forest ecosystem [6, 12].

Detritivores, such as earthworms and many coleopteran larvae, inhabiting organic surface layers that are highly contaminated with radionuclides are important decomposers in ecosystem material cycles and may also contribute to the movement of ^137^Cs in the forest floor [13, 14]. Detritivores may play a major role in ^137^Cs uptake from the forest floor into forest ecosystems because they are consumed by various animals at higher trophic levels, such as birds, mammals, and fish. In forest insect communities, detritivorous insects have higher ^137^Cs concentrations than herbivorous insects [15]. In headwater streams, fish consume both aquatic and terrestrial insects. Thus, they can become contaminated with ^137^Cs through consuming ^137^Cs-contaminated detritivorous insects [16]. However, some studies that assessed the transfer of ^137^Cs from litter to detritivores suggested that the bioavailability of ^137^Cs in these organisms is extremely low. For example, 95% of ^137^Cs in earthworms is present in the digestive tract and is mostly excreted without being transferred to other parts of the body tissues [13]. Further, the leachability of ^137^Cs, or its bioavailability, in substrates is determined by its chemical form and has been evaluated in extraction experiments [11, 12, 17]. Manaka et al. (2019, 2020) reported that relatively mobile ^137^Cs in organic matter rapidly decreased in the months following the Fukushima Daiichi Nuclear Power Plant accident [12] but its proportion has since remained constant [11]. Therefore, ^137^Cs bioavailability in litter and uptake into detritivores should be quantitatively evaluated to understand their role in ^137^Cs dynamics in forest ecosystems and the contamination of organisms of higher trophic levels, such as fish. To the best of our knowledge, comparable ^137^Cs absorption has not been estimated for other large soil invertebrates.

Thus, this study investigated the transfer of ^137^Cs into larvae of flower chafer (*Protaetia orientalis*) (Coleoptera: Scarabaeidae), which is commonly distributed throughout Japan. Among several species tested in preliminary experiments, *P*. *orientalis* was selected for the study due to its prevalence as a dominant detritivore litter feeder in the forests of Fukushima and its demonstrated suitability for laboratory breeding. The larvae feed on a mixture of decomposed leaf litter and fermented wood approximately 10 months before imago emergence. First, we conducted breeding experiments to evaluate the uptake and excretion of ^137^Cs by flower chafer larvae. ^137^Cs distribution in the muscle, digestive tract, and skin was investigated via dissection. Additionally, the ^137^Cs absorption rate at which ^137^Cs in the litter was transferred to the larval tissue, was calculated. ^137^Cs excretion in flower chafer larvae was investigated using elimination experiments. Second, sequential extraction was conducted to determine the proportion of bioavailable ^137^Cs within the litter. Comparison with the absorption rates from the breeding experiments allowed us to investigate how much bioavailable ^137^Cs in the sequential extractions was transferred to the larvae. Furthermore, by comparing the sequential extraction results between the litter and feces, we revealed the physicochemical forms of ^137^Cs in the litter that the larvae could absorb through digestion.

## Materials and methods

### Litter preparation

The decomposed litter used in the rearing experiment was collected in August 2020 from a deciduous broadleaf forest located in the upper reaches of the Ota River in Minamisōma City, Fukushima Prefecture. The site is approximately 30 km away from Tokyo Electric Power Company’s Fukushima Daiichi Nuclear Power Plant. This site is the forest where we have been studying the transfer of ^137^Cs from aquatic and terrestrial insects to fish that consume them [18]. The litter was collected from relatively flat areas alongside headwater stream, where broadleaf litter accumulated. Dry streams or slope area were excluded to minimize the effects of wash-off due to water flow. The decomposed litter was obtained from the partially decomposed organic layer (F-H layer, including fermentation and humus sublayers) after removing fresh litter from the surface. Sampling of approximately 1.5 kg of litter was performed at 10 different locations and the collected litter was mixed. The litter was brought to the laboratory, dried in an oven at 60 °C for 7 d, ground and homogenized using an impact mill (IFM-800DG; Iwatani Ltd., Tokyo, Japan), and sieved through a 1-mm mesh sieve. Because there is a restriction on the quantity of radiocesium material that can be brought into the laboratory according to the safety management regulations for sample handling personnel established by the institute, the litter was used after mixing with ^137^Cs-free commercially available substrate, which was made from powdered decayed oak wood (spawning first; Fortech Ltd., Wakayama, Japan) at a ratio of 1:1.

### Preparation of flower chafer

**Fig 1:**
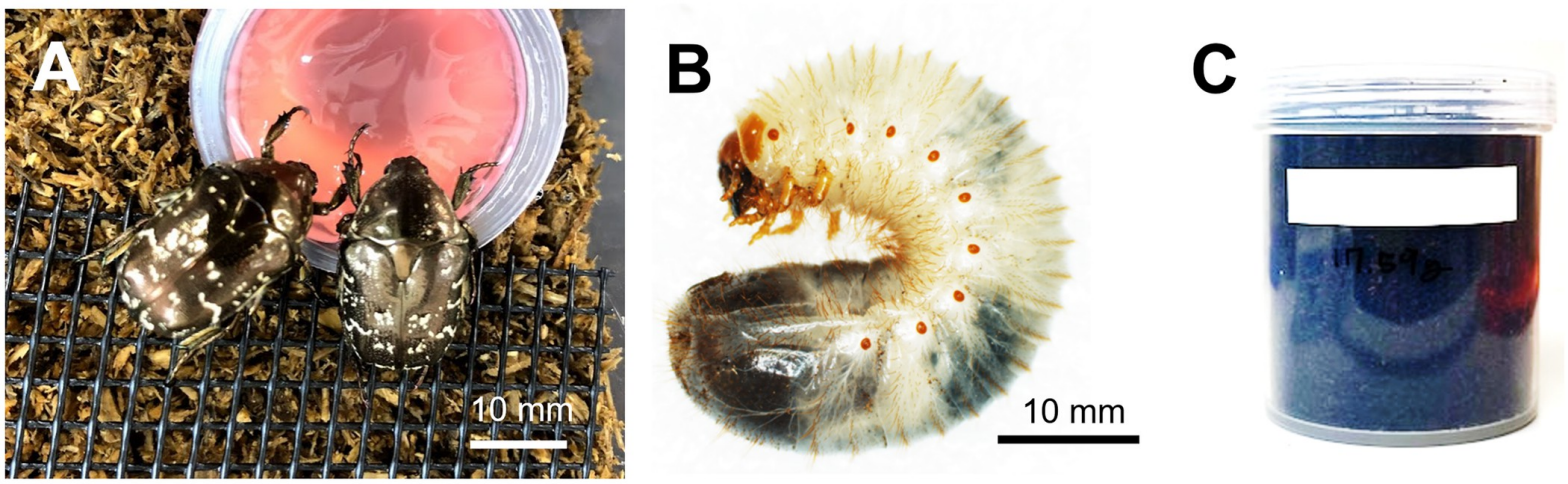
Rearing experiment. Pictures of (A) Imago and (B) larva of flower chafer, and (C) the U-8 container used for the rearing experiment.

We collected flower chafer imagines from May to September 2020 using traps at the institution property in Miharu Town, Fukushima Prefecture (Fig 1A). *Protaetia orientalis* is a common and known pest species [19]. It is not listed as a nationally endangered species under the Law for the Conservation of Endangered Species of Wild Flora and Fauna of Japan (https://www.env.go.jp/en/nature/biodiv/law.html). Therefore, permissions are not required for its collection and experimentation in the study area. The collected imagines were placed in a cage with a commercial ^137^Cs-free substrate for oviposition and their offspring were used for the breeding experiment. The larvae were reared for 3–5 months at 20 °C. Subsequently, the third and last instar larvae were used for the rearing experiment (Fig 1B).

### Breeding experiment

**Fig 2:**
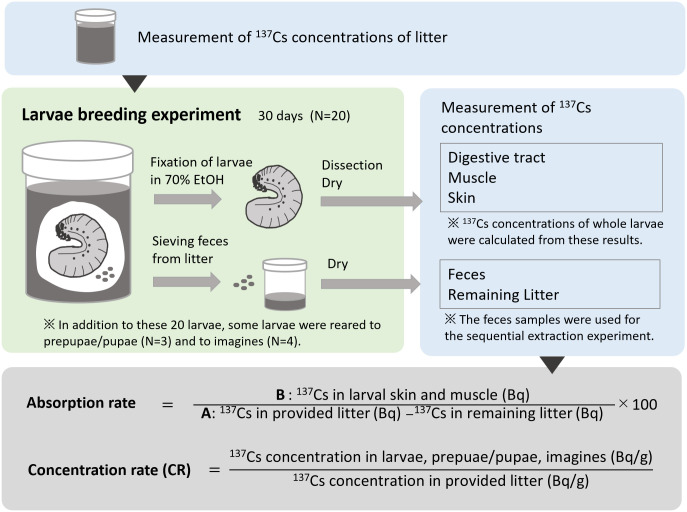
Flow chart of the breeding experiment.

The design of the breeding experiment is shown in Fig 2. Before starting the breeding experiment, 250 g of litter moistened with water was placed in a plastic container (Fig 1C, U8 container, d = 50 mm; h = 62 mm) and the ^137^Cs activity concentration of the litter was measured. Twenty third-instar larvae were reared for 30 d, with one larva in each U8 container kept in a laboratory under dark conditions at 20 °C. After one month, the larvae were removed from the U8 containers and fixed in 70% ethanol. The fixed larvae were cleaned by removing body surface contaminants using a brush. Subsequently, their muscle, skin, and digestive tract were dissected using dissecting scissors and the dissected body parts were dried at 60 °C for approximately 2 d. We prepared samples of each individual larvae, obtaining a total of 20 samples of each body part. After the dry weights were measured using a balance (XSE205 DualRange; Mettler Toledo Ltd., Tokyo, Japan), samples were cut into small pieces using dissecting scissors and stored in flat-bottom test tubes (diameter = 15 mm and height = 57 mm) until subsequent germanium (Ge) *γ*-ray spectrometry analyses. The larval feces left in pellet form in the U8 container were separated from the remaining leaf litter using a sieve with a mesh size of 1 mm. The feces and remaining leaf litter were dried at 60 °C for approximately 2 d, the dry weights were measured and homogenized, and the samples were stored in U8 containers until Ge *γ*-ray spectrometry analyses. Some larvae were reared until they reached the imago stage in the U8 containers by using the same litter; however, many larvae died before reaching the imago stage, resulting in four samples for prepupae/pupae and three samples of imagines.

### Estimating the biological half-life of ^137^Cs

Approximately 60 third-instar larvae were reared for one month using the same litter containing ^137^Cs as used in the breeding experiment in three cages (33 × 23 × 19 cm, 20 larvae per cage). They were then moved to a commercial ^137^Cs-free substrate in three cages (33 × 23 × 19 cm, 20 larvae per cage) to evaluate the elimination of ^137^Cs from the larvae. Subsequently, the larvae of six individuals were preserved in 70% ethanol for 0, 1, 3, 6, 12, 24, 48, 96, and 192 h. The larvae were then dissected, dried, weighed, homogenized, and stored in flat-bottom test tubes until Ge *γ*-ray spectrometry analyses, following the same procedures as those used for the breeding experiments.

The biological half-lives of ^137^Cs in flower chafer larvae were estimated for the digestive tract, skin, and muscle tissues separately because different ^137^Cs elimination rates were assumed for each part [13, 20, 21]. ^137^Cs elimination was described using the following single- and two-component exponential models:

$$\text{Single-component}\;\text{model}:A_{t}=A_{0}e^{-\lambda t}$$ (1)

where A_0_ = initial ^137^Cs activity concentration (Bq/g), A_t_ = ^137^Cs activity concentration at time t (days from the initial date of the larvae moving to ^137^Cs-free litter) (Bq/g), and λ = elimination rate constants (d^-1^).

$$\text{Two-component}\;\text{model}:A_{t}=A_{1}e^{-\lambda_{1}t}+A_{2}e^{-\lambda_{2}t}$$ (2)

where A_1,_ A_2_ = initial ^137^Cs activity concentration of the first and second components, respectively (Bq/g), A_t_ = ^137^Cs activity concentration at time t (Bq/g), and λ_1_ and λ_2_ = elimination rate constants (d^-1^) for each component. A two-component model has been used to model cases wherein the contaminant initially declines rapidly, followed by a gradual rate of decline [13, 20–22]. The parameters for these equations were estimated by performing a non-linear least-squares method using the “nls” function of R ver. 4. 3. 3, a programming language for statistical computing supported by the R Core Team and the R Foundation for Statistical Computing [23]. The AIC was compared between the estimated single and two-component model and the two-component model was selected because it had a smaller AIC value in both cases: digestive tract: AIC_single-component model_ = 104.3 and AIC_two-component model_ = 59.4 and skin and muscle tissues: AIC_single-component model_ = 48.0 and AIC_two-component model_ = 43.4). Using the rapid and slow decreasing rate constants, λ_1_ and λ_2_, of the two-component model, the biological half-lives (T_b_) were calculated.

### Sequential extraction of ^137^Cs

After larval consumption, the sequential extraction method was adopted for the litter and fecal pellets. Twelve replicates of litter were subsampled from the litter prepared for the rearing experiment and 12 replicates of fecal pellets were prepared from 12 of the 20 individuals used for the rearing experiments. The homogenized litter and fecal samples after Ge γ-ray spectrometry analyses were dried at 60 °C for approximately 1 d before subsample.

Sequential extraction has been applied to evaluate the chemical forms of ^137^Cs in soil, sediments, and litter [11, 12, 17, 24]. We conducted a ^137^Cs extraction experiment based on Takechi et al. (2020); however, we added a water-soluble extraction step as a first step. We sequentially extracted ^137^Cs, which exists in a (1) water-soluble form, (2) ion-exchangeable form (^137^Cs that can be exchanged by ammonium ions), (3) oxide form (^137^Cs that is bound to iron and manganese oxides and extracted by reductive dissolution), (4) organic form (^137^Cs that is bound to organic matter and extracted by oxidizing and decomposing organic matter), and (5) fixed form (^137^Cs that is trapped in minerals and is scarcely dissolved). The litter and fecal pellet samples were used for the following extraction procedure after determining ^137^Cs activity concentration by Ge *γ*-ray spectrometry analyses. The steps can be described in detail below:

Step (1): Water-soluble form. Three grams (dry weight) of each sample were placed in a centrifuge tube and 30 mL of pure water was added. Three fecal pellet samples weighed less than 3 g (2.87–2.96 g) owing to insufficient sample volume. In these cases, the solvent was added at a ratio of 1 (sample):10 (solvent). The tube was stirred (150 rpm) at room temperature (25 °C) for 16 h using shaking incubators (PRAS, 12-R-FF, Preci Co., Ltd.) and then centrifuged at 7,931 rpm for 30 min (7000, KUBOTA) to separate the supernatant and residue. The supernatant was spontaneously filtered through a filter paper (Whatman 42, Global Life Science Technologies Japan K.K., pore size 2.5 μm) and the extract acquired after filtering was transferred to a U8 container. The residue was rinsed with 30 mL pure water, centrifuged at 7,931 rpm for 30 min, and the resultant supernatant was discarded. In each subsequent extraction, the residue was rinsed with the same volume of pure water as the extraction solution added in each step.
Step (2): Ion-exchangeable form. The residue from the previous step was mixed with 30 mL of 1 mol/L ammonium nitrate solution (1 mol/L NH_4_NO_3_). The resultant suspension was stirred, centrifuged, and filtered, and the acquired extract was separated under the same conditions as described in Step (1).
Step (3): Oxide form. The residue of the previous step was mixed with 60 mL of 0.5 mol/L hydroxyl amine hydrochloride (HONH_2_-HCl, containing 2.5% v/v 2.0 mol/L HNO_3_). The suspension was stirred, centrifuged, and filtered, and the extract was separated under the same conditions as described in Step (1).
Step (4): Organic form. The residue of the previous step was mixed with 12 mL of hydrogen peroxide (H_2_O_2_ 36%), adjusted to pH 2.0 using 1% HNO_3,_ and left at room temperature (20°C) for approximately 1 h until the reaction settled. The container (with a loose lid) containing the sample was placed in a thermostatic bath (Thermominder, SX-10N, TAITEC) at 85 °C and heated for 1 h while shaking it occasionally. Thereafter, the liquid was heated without a lid until the volume was less than 4 mL. This process was repeated two times. After the sample had cooled to room temperature, 36 mL of 1.0 mol/L NH_4_NO_3_ adjusted to pH 2.0 using concentrated nitric acid was added. The suspension was stirred, centrifuged, and filtered, and the extract was separated under the same conditions as described in Step (1).
Step (5): Residuals. Finally, the residue was transferred to a U8 container and thoroughly dried at 105 °C, and the dry weights were measured. The extracts obtained from Steps (1)–(4) were transferred to a U8 container and weighed.

### ^137^Cs measurements

The radioactivity of the flower chafer samples was measured using a high-purity germanium (HPGe) coaxial detector (Canberra GCW6023; Mirion Technologies, Canberra Ltd., Tokyo, Japan). The leaf litter and fecal samples from the breeding experiment and the extract and residue from the sequential extraction experiment were stored in U8 containers, and ^137^Cs activity concentrations were measured using HPGe (Canberra GCW6023; Mirion Technologies, Canberra Ltd., Tokyo, Japan, and SEG-EMS GEM 30–70; SEIKO EG&G Ltd., Tokyo, Japan). Most samples were measured for <10% of counting error, except for small quantities of dissected flower chafer samples in flat-bottomed test tubes, which were measured for <15% of counting error. The minimum detectable amount of ^137^Cs above the detection limit was about 0.07 Bq in the well-type HPGe coaxial detector. The standardized mixed sources (^134^Cs and ^137^Cs) for calibrating the detectors were MX035 (Japan Radioisotope Association, Tokyo, Japan) for the well-type HPGe coaxial detectors and MX033U8PP (Japan Radioisotope Association, Tokyo, Japan) for the U8 container. The standardized mixed sources were measured with varying filling quantities. Measurement efficiencies for ^134^Cs (604.66 and 795.76 keV) and ^137^Cs (661.66 keV) were obtained, and three efficiency calibration curves were created for different filling heights. The concentration of ^137^Cs was then calculated using the comparative method based on these curves. Gamma Studio software (SEIKO EG&G, Tokyo, Japan) and Spectrum Explorer (Mirion Technologies, Canberra Ltd., Tokyo, Japan) were used to analyze the *γ*-ray spectra. The sample activities were corrected for radioactive decay until sample collection. ^137^Cs activity concentrations were expressed as ^137^Cs concentrations on a dry weight basis. S1 and S2 Tables show the ^137^Cs activity concentrations (Bq/kg) and total ^137^Cs (Bq) of the breeding experiment and sequential extraction experiment, respectively.

### Statistical analysis and calculation of assimilation rate and concentration ratio

For the breeding experiment, the distribution of both ^137^Cs concentrations and log-transformed ^137^Cs concentrations were not normal by the Shapiro-Wilk normality test and not equivalent across samples by the Levene’s Test for Homogeneity of Variance. Therefore, multiple comparisons were made using pairwise Wilcoxon rank-sum tests for ^137^Cs concentrations in litter before breeding, feces, dissected tissues of flower chafers (digestive tract, skin, and muscle), and whole-body larvae with Bonferroni adjustments for *p* values. The ^137^Cs concentrations in whole-body larvae were calculated using the weight and concentration of each tissue in the dissection experiment assuming that the total ^137^Cs in whole-body larvae is equivalent to the sum of ^137^Cs in each body part. The absorption rate of ^137^Cs in flower chafer larvae during the breeding experiment was calculated for each individual (Fig 2). The total ^137^Cs in the litter consumed by the larvae was calculated by subtracting the total ^137^Cs in the remaining litter after breeding from the total ^137^Cs in the provided litter (A). The total ^137^Cs assimilated in the larval tissue was calculated as the sum of ^137^Cs in the larval skin and muscle (B). The absorption rate at which ^137^Cs in the diet was transferred to flower chafer larval tissue at the end of the experiment was calculated as (B/A) × 100. ^137^Cs concentrations in whole-body larvae, prepupae/pupae, and imagines were compared using the pairwise Wilcoxon rank-sum test. Additionally, the concentration ratio (CR) was calculated as follows: CR = ^137^Cs concentration in flower chafer (Bq/g) / ^137^Cs concentration in litter (Bq/g) for whole-body larvae, prepupae/pupae, and imagines.

Based on the results of the sequential extraction experiment, the bioavailable fraction of ^137^Cs (percentage of the sum of water-soluble, exchangeable, oxide, and organic forms to the total ^137^Cs contained in the sample) between the litter and feces excreted from larvae was compared using pairwise Wilcoxon rank-sum tests. The quantity of extracted ^137^Cs from the sample per gram in water-soluble, exchangeable, oxide, and organic forms and the remaining ^137^Cs were also compared between the litter and feces. Because the distributions of the log-transformed total quantities of the extract were not normal and did not have equivalence variance across samples, we applied pairwise Wilcoxon rank-sum tests. All statistical analyses and calculations were performed using R ver. 4.3.1. [23].

## Results and discussion

### Uptake and absorption of ^137^Cs into flower chafer larvae

**Fig 3:**
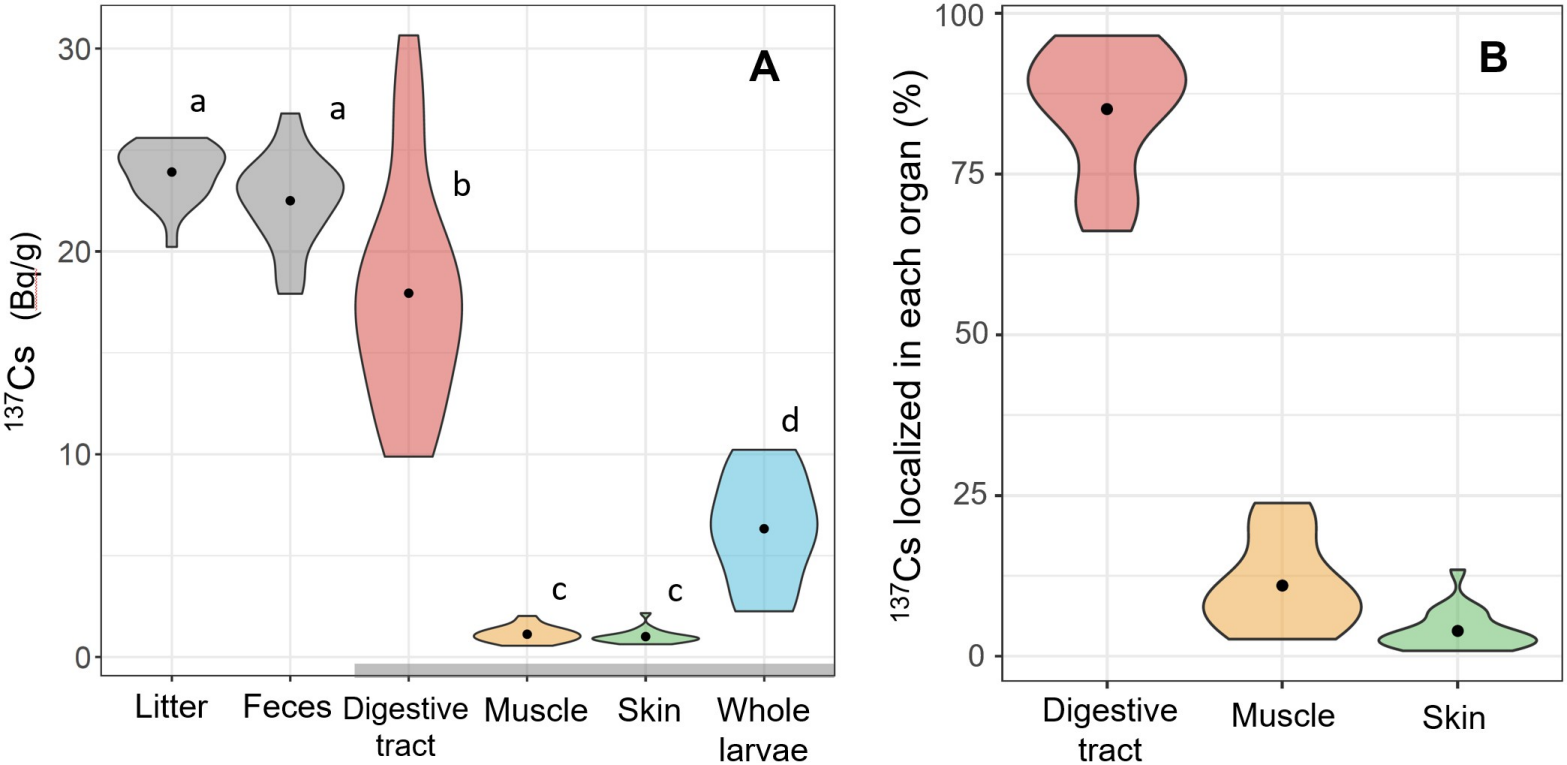
Concentrations of ^137^Cs concentrations in flower chafer larvae. (A)^137^Cs concentrations in flower chafer larvae after the breeding experiment. Litter (provided as diet), and feces, digestive tract, skin, muscle, and whole-body of larvae are shown (N = 20). Different letters beside each box indicate significant differences in the ^137^Cs concentrations based on the Wilcoxon rank-sum tests. (B) Percentage of ^137^Cs distributed in each organ of larvae for total ^137^Cs in flower chafer larvae (N = 20). Each violin represents the distribution of an individual measure, and the dots represent the means.

The results of the dissection experiment showed that the ^137^Cs concentration in the digestive tract of the larvae was significantly higher than that in the skin and muscle tissues (*p* < 0.001, Fig 3A). The calculated distribution of ^137^Cs in larvae showed that most ^137^Cs was localized in the digestive tract of the larvae (85.1 ± 9.7% of the total ^137^Cs), whereas the muscle and skin included only 10.9 ± 6.9% and 3.9 ± 3.1% of the total ^137^Cs, respectively (Fig 3B). This ^137^Cs distribution was comparable to that of earthworms showing 95% of the total ^137^Cs in the digestive tract [13]. Most ^137^Cs concentrations in detritivorous aquatic insects also have been described by their digestive tract contents, the removal of which has been observed to significantly reduce ^137^Cs concentrations [20, 25].

No statistically significant changes in ^137^Cs concentrations were detected between the litter and feces (*p* = 0.25, Fig 3A), possibly because the ^137^Cs decrease due to transfer from litter to larvae was marginal. Additionally, fecal samples may have been contaminated with the surrounding litter powders when the larvae were discharged, which is indicated by the ^137^Cs concentrations in feces being significantly higher than those in the digestive tract contents obtained by dissection (*p* = 0.018).

The localization of ^137^Cs in the digestive tract indicates that most of the ^137^Cs ingested by flower chafer larvae may be excreted in feces, with little ^137^Cs absorbed into the body tissues. Moreover, the ^137^Cs absorption rate estimated in this study was extremely low. The total ^137^Cs in the litter consumed during one month of breeding (A) was 132.0 ± 49.8 Bq and that transferred to the body tissues of muscle and skin (B) was 0.42 ± 0.1 Bq; thus, the absorption rate was 0.39 ± 0.2%. Few comparable studies have investigated ^137^Cs absorption by soil macroinvertebrates [13]. However, ^137^Cs absorption in fish has been estimated via feeding experiments, in which fish were fed with radiocesium-labeled diets; notably, ^137^Cs absorption varied greatly depending on the food items. ^137^Cs absorption in brown trout was 55% from Chironormidae sp. Larvae, 48% from freshwater amphipods, 76% from freshwater snails, 23% from Ephemeroptera sp. Larvae, 82% from zooplankton, and 66% from brown trout muscle [26]. Much lower ^137^Cs absorption in bluegill was reported from detritus (3%) and sediment-fed *Chironomus* larvae (7%–16%), indicating that fish can absorb most of the ^137^Cs associated with tissues and absorb marginal quantities of ^137^Cs from detritus and clay in *Chironomus* larvae [27]. ^137^Cs absorption in flower chafer larvae in this study was lower than that in fish, indicating significantly lower absorption rates for soil macroinvertebrates from detritus containing clay minerals. ^137^Cs absorption can be affected by the physicochemical forms of ^137^Cs in the litter and the quantity of clay minerals; however, future studies are needed to clarify this relationship.

By rearing flower chafers to the imago stage, ^137^Cs concentrations were substantially reduced throughout the developmental stages (Table 1). Compared to the concentrations in whole-body larvae, ^137^Cs concentrations were reduced by 50% in prepupae/pupae (*p* = 0.06, marginal due to small sample size) and 72% in imagines (*p* < 0.01). The larva discharges most of its digestive tract content by purging the gut before pupation. Subsequently, the digestive tract structure of scarab beetles completely changes during the pupal stage, and the cuticle of the digestive tract and the remaining contents are excreted during eclosion [28]. These metamorphosis processes may eliminate ^137^Cs from the digestive tract. The ^137^Cs concentrations in imagines were approximately equal to those assimilated in the skin and muscle tissues of the larvae, suggesting that the imago ^137^Cs concentration was determined by the ^137^Cs assimilated into the tissues during the larval stage (Table 1). The calculated whole body ^137^Cs CRs for larvae, prepupa/pupa, and imago were 0.27 ± 0.11, 0.13 ± 0.03, and 0.06 ± 0.02, respectively (Table 1). These CR values were almost consistent with the CR value (0.13 ± 0.10) determined previously for forest detritivorous insects (all CRs were calculated for the imago stage) [15], thus, confirming that the transferred ^137^Cs concentration evaluated in our breeding experiment was within the range observed in the field.

**Table 1:** ^137^Cs concentrations and concentration ratios for flower chafer larvae at each developmental stage. Standard deviations are shown in parentheses.

Category	^137^Cs (Bq/g-dry)	N	Concentration ratio
Whole body larva	6.32 ± 2.6	20	0.27 ± 0.11
Larval muscle	1.12 ±0.4	20	–
Larval skin	1.00 ±0.3	20	–
Prepupa / Pupa	3.21 ±0.6	4	0.13 ± 0.03
Imago	1.76 ±0.6	3	0.06 ± 0.02

### Estimation of the biological half-life of ^137^Cs

**Fig 4:**
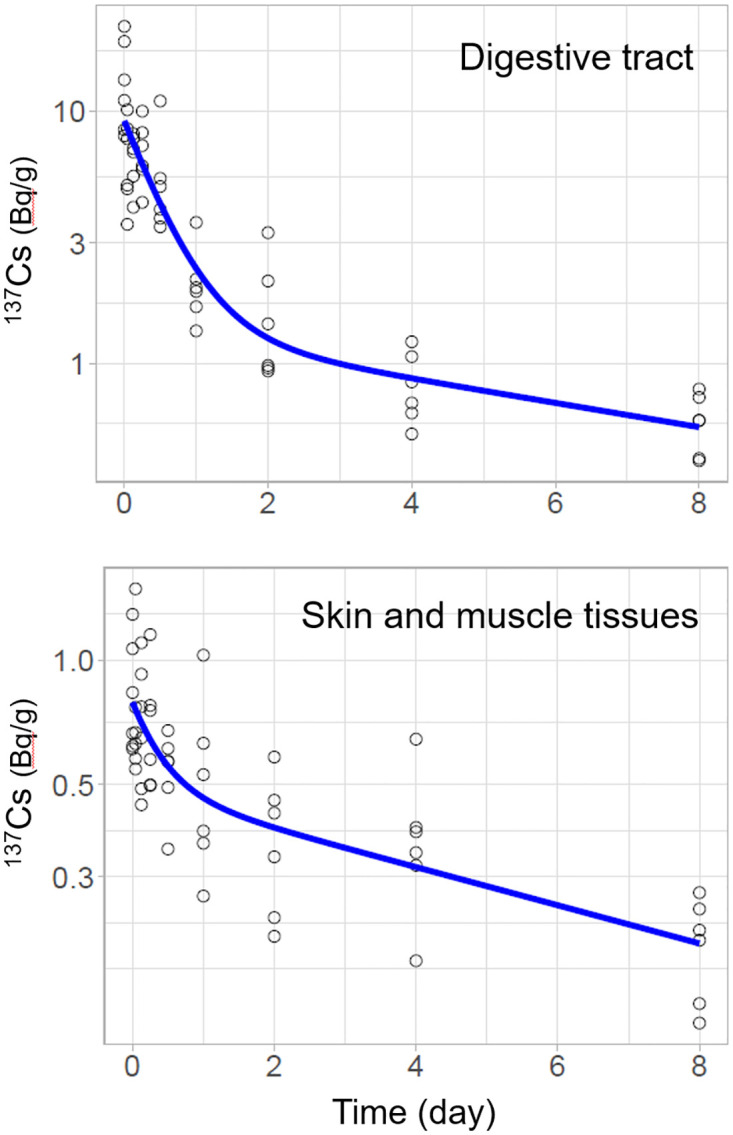
Elimination of ^137^Cs from flower chafer larvae. The lines represent the prediction of the fitted two-component model (Table 2).

In the elimination experiment, ^137^Cs concentrations decreased at different rates in the digestive tract, muscles, and skin tissues (Fig 4; Table 2). ^137^Cs in the digestive tract was rapidly eliminated during the first 2 d. This result was consistent with a previous finding that reported that the contents were completely excreted from the digestive tract within 47 h in third-instar scarab larvae (*Trypoxylus dichotomus*) [29]. After 2 d, when the digestive tract contents were assumed to have been replaced by clean litter, ^137^Cs in the digestive tract remained and decayed slowly. This can be attributed to litter powder trapped by the walls of the digestive tract. Although ^137^Cs in the digestive tract gradually decreased, it was expected to remain in the tract until it was completely excreted by pupation and eclosion, as has been shown in breeding experiments up to the imago stage. The estimated biological half-life of ^137^Cs in the fast component by excretion of digestive tract contents (0.26–0.64 d; T_b1_ for digestive tract in Table 2) was slightly slower than those estimated for earthworms (0.10–0.33 d; summarized in Table 1 in Tanaka et al. 2018) and Trichopteran larvae (0.22–0.37 d; Fujino et al. 2018).

**Table 2:** Estimated parameters for the two-component exponential model of ^137^Cs concentrations for flower chafer larvae. For biological half-life, T_b_, 95% confidence intervals are shown in parentheses.

Body part	A_1_ (Bq/g-dry)	λ_1_	A_2_ (Bq/g-dry)	λ_2_	T_b1_ (day)	T_b2_ (day)
Digestive tract	7.82	1.90	1.35	0.11	0.36 (0.26, 0.64)	6.27 (3.35, 48.30)
Muscle and skin tissues	0.30	2.33	0.48	0.11	0.30 (0.11, 0.47)	6.54 (4.01, 17.70)

Contrastingly, ^137^Cs in skin and muscle tissues decreased rapidly during the first day and a relatively constant decrease of ^137^Cs followed until Day 8. The two-component model indicated that different pools of ^137^Cs were likely involved in ^137^Cs elimination [21, 30]. The fast component may be small pools of ^137^Cs that are easily discharged within 1 d and the slow component may be ^137^Cs that is assimilated into tissues and gradually discharged. The estimated biological half-life of the slow component (4.0–17.6 d; T_b2_ for muscle and skin in Table 2) was relatively faster than that estimated in earthworms (15–54 d; summarized in Table 1 in Tanaka et al. 2018), although the direct comparisons were difficult due to the short experimental period of this study.

^137^Cs whole-body burdens have been used to estimate the half-life in earthworms [13] and invertebrates [20, 21, 31]. In these estimated two-component models, short-lived and long-lived components were interpreted to represent gut clearance and physiological clearance of assimilated ^137^Cs, respectively. However, our dissection experiment revealed ^137^Cs kinetics more comprehensively; particularly, digestive tract and physiological clearance were represented by a two-component model respectively consisting of multiple ^137^Cs pools.

### Bioavailable ^137^Cs by sequential extractions

**Fig 5:**
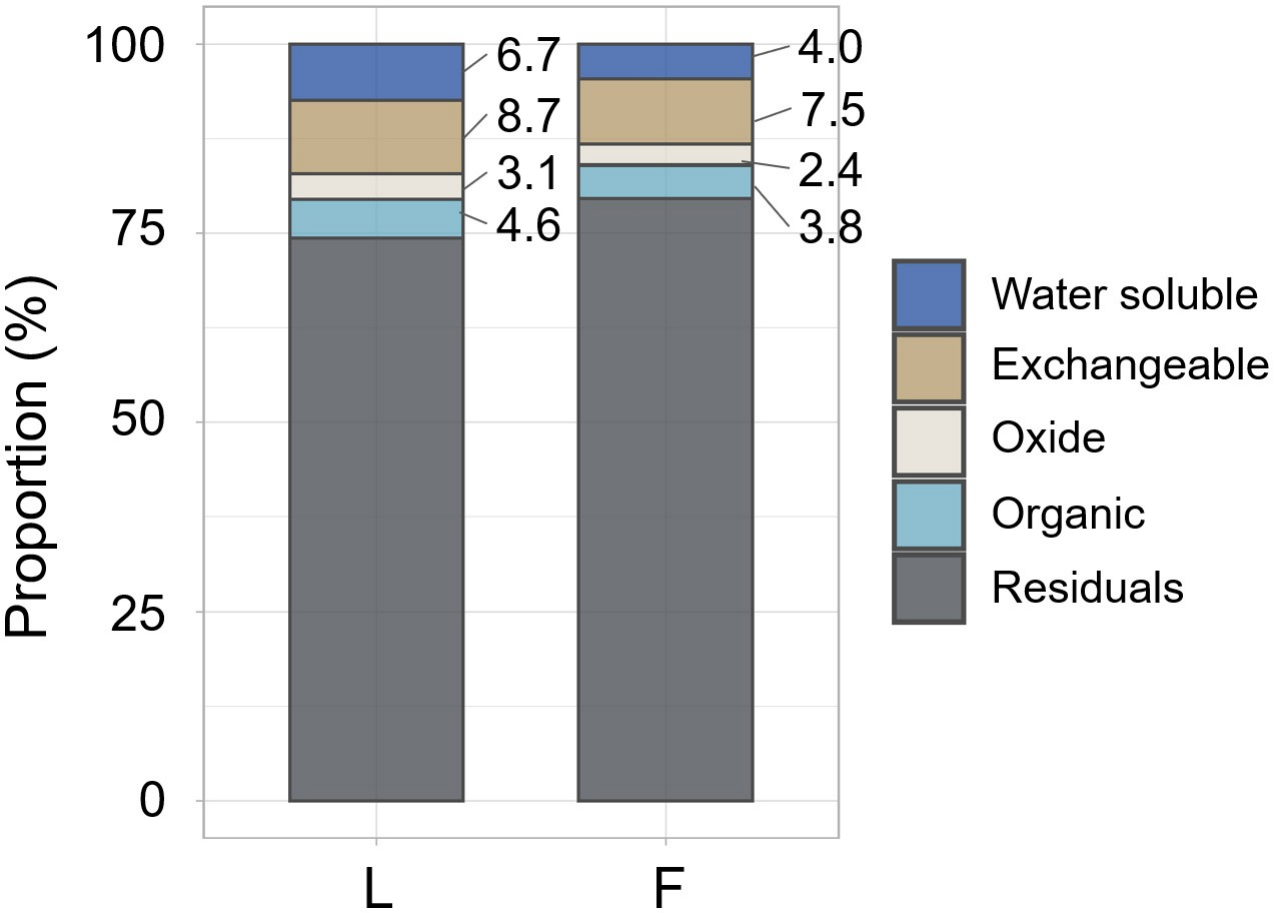
Proportion of different forms of ^137^Cs in litter (L) and feces (F). The proportions shown are the means of 12 samples for litter and feces.

We calculated the recovery rate by dividing the sum of ^137^Cs extracted in each fraction by the total ^137^Cs present in the pre-extraction samples. This yielded a recovery rate of 94.7 ± 7.9%, confirming the validity of the sequential extraction analysis of this study. Fig 5 shows the results of the sequential extraction of litter and feces after conducting the breeding experiment. Because ^137^Cs dissolution primarily occurs by ion exchange, reductive dissolution of Fe and Mn oxides, and microbial degradation of organic matter, these fractions (water-soluble, exchangeable, oxide, and organic forms) were considered as the bioavailable fraction [17].

**Fig 6:**
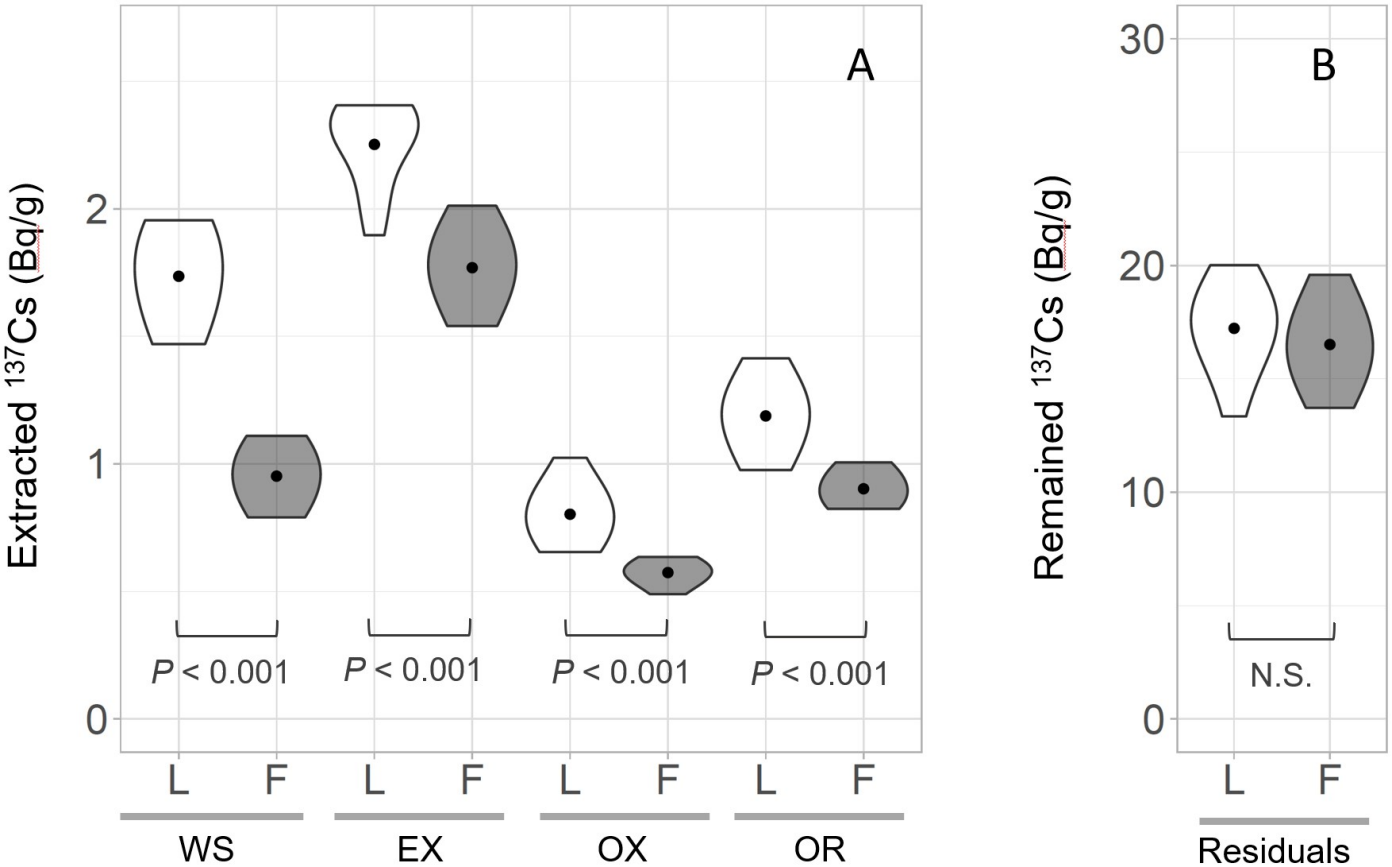
Extracted bioavailable fraction of ^137^Cs. Extracted bioavailable fraction of ^137^Cs from (A) water-soluble (WS), exchangeable (EX), oxidized (OX), and OR (organic) forms from litter (L) and feces (F) (N = 12). (B) ^137^Cs remaining in residuals. Each violin represents the distribution of an individual measure, and the dots show the means. The *p*-values show the result of the Wilcoxon rank-sum tests.

The previous study conducting the sequential extraction experiments of litter in Fukushima reported that relatively mobile ^137^Cs remained in constant proportions from 2012 to 2019, which were assumed to be co-extracted with soluble fats, waxes, oils, holocellulose, and acid-soluble lignin [11]. These forms of ^137^Cs may be extracted as bioavailable ^137^Cs in this experiment, although direct comparison is not possible due to differences in extraction solvents. Litter digestion by flower chafer significantly reduced bioavailable fraction from 23.2 ± 2.3% in litter to 17.7 ± 2.5% in feces (*p* < 0.001). Additionally, the quantity of extracted ^137^Cs was significantly lower in the feces than in the litter for all forms of bioavailable ^137^Cs (Fig 6A); the reduction was 45%, 22%, 29%, and 24% for water-soluble, exchangeable, oxidized, and organic forms, respectively. This suggested that litter feeding by flower chafer larvae may absorb ^137^Cs through the digestive process not only in an easy-elution form (water soluble and exchangeable) but also in oxide and organic forms.

Scarab larvae consume cellulose from various sources, including plant roots, soil organic matter, and decaying wood, and extract nutrients and energy from these sources [32]. The degradation mechanism of these components in the digestive tract has been well-studied, especially in the scarab larvae of *Pachnoda ephippiata* [32, 33]. Particularly, their midgut is highly alkaline (pH 12) and the most easily digestible proteins are mobilized and absorbed by endogenous proteinases. The digestive tract contents are then passed into a hindgut fermentation chamber containing diverse microorganisms, which degrade cellulose under anaerobic conditions. The digestive system of scarab larvae may promote ^137^Cs elution and absorption in oxide and organic forms present in the litter. In contrast, in our study, digestion did not reduce the quantity of residual ^137^Cs in the feces compared to that in the litter (Fig 6B). More than 70% of the ^137^Cs remained as residuals, which may include ^137^Cs fixed in clay minerals [6, 7, 34]. In addition, the distribution of radiocesium-bearing microparticles (CsMPs) has been reported for the Ota River watershed where the litter was collected; therefore, CsMPs can also contribute to ^137^Cs in the residuals [18]. The ^137^Cs in clay minerals and CsMPs is assumed to be unavailable for biological uptake during digestion. Further investigation for the quantity of clay minerals and CsMPs present in the litter is needed because it is likely to have a significant impact on both the results of the sequential extraction and the ^137^Cs absorption rate in flower chafer larvae.

The digestion process by detritivores potentially influences the bioavailability of ^137^Cs in the litter via decomposition [14, 35, 36]. Our results showed that digestion by larvae reduced the bioavailable fraction of ^137^Cs in the litter. This was contrary to the findings of previous studies that have suggested that digestion of litter by scarab larvae (*Trypoxylus dichotomus*) significantly increases the exchangeable fraction of KCl in the litter [14]. This inconsistency could be attributed to differences in the litter provided. Ishii et al. (2018) fed larvae with intact litter and concluded that physical decomposition increased ^137^Cs elution in feces. However, the larvae in our experiment were fed with finely crushed litter; thus, the effect of physical decomposition was marginal, but the chemical digestion process may have reduced the bioavailable ^137^Cs in the feces. The effect of the ingestion of flower chafer larvae on the bioavailability of ^137^Cs in intact litter under natural conditions remains unclear. Therefore, further studies are required to evaluate the impact of feeding by detritivores on the bioavailability of ^137^Cs in forest floor litter.

The low bioavailability of ^137^Cs in the litter observed in this study was consistent with earlier observations that reported low absorption ^137^Cs in the detritus of earthworms [13]. However, detritus feeders may play an important role in ^137^Cs input into forest ecosystems by extracting the bioavailable fraction of ^137^Cs stock that is found in abandance on the forest floor. Furthermore,^137^Cs in the skin and muscle tissues of detritivores is probably present in a highly bioavailable form that is susceptible to absorption by predators, such as fish. Therefore, it is likely that more bioavailable ^137^Cs accumulates at higher trophic levels in the food web. In the aquatic food web, the sequential extraction results indicated that the ratio of easy-elution forms (exchangeable and carbonate forms) was higher in planktivorous fish (almost 100%) than in plankton (40%–80%) [37]. Thus, estimating the bioavailability of ^137^Cs in detritivore and higher trophic organisms would be useful for understanding ^137^Cs dynamics through the detrital food chain.

Furthermore, future work to estimate the quantity of ^137^Cs uptake by detritivores against stock in the forest floor is needed to elucidate the contribution of detritivores to the contamination of organisms in the forest ecosystem. In addition, the bioavailability and absorption rates of ^137^Cs in forest litter may be influenced by factors such as tree species and the quantity of fixed ^137^Cs in clay minerals. Therefore, the ^137^Cs availability and absorption rates estimated in this study may not fully represent the broad contaminated area across Fukushima Prefecture. Estimating these parameters among different regions with varying tree species and other detritivorous insect species, would contribute to a more comprehensive understanding of the role of detritivores in the dynamics of ^137^Cs in forest ecosystems.

## Conclusion

Our study quantitatively demonstrated the low bioavailability of ^137^Cs in forest floor litter and lower absorption rates of ^137^Cs in flower chafer larvae. Although 23% of the ^137^Cs in the litter was in the bioavailable form as evaluated during the sequential extraction, the breeding experiment showed that only 0.4% of ^137^Cs was absorbed into the larvae. Flower chafer larvae absorbed ^137^Cs through digestion, not only in an easy-elution form (water-soluble and exchangeable) but also in oxide and organic forms. Our study indicated that detritivores may play a role in extracting the bioavailable fraction of ^137^Cs remaining in large quantities on the forest floor. Further studies are needed to evaluate the contribution of detritivores to ^137^Cs transfer into organisms at higher trophic levels and forest ecosystems.

## Supporting information

S1 Table (XLSX)

S2 Table (XLSX)
